# Chickpea transcription factor CaTLP1 interacts with protein kinases, modulates ROS accumulation and promotes ABA-mediated stomatal closure

**DOI:** 10.1038/srep38121

**Published:** 2016-12-09

**Authors:** Vijay Wardhan, Aarti Pandey, Subhra Chakraborty, Niranjan Chakraborty

**Affiliations:** 1 National Institute of Plant Genome Research, Jawaharlal Nehru University Campus, Aruna Asaf Ali Marg, New Delhi-110067, India

## Abstract

Tubby and Tubby-like proteins (TLPs), in mammals, play critical roles in neural development, while its function in plants is largely unknown. We previously demonstrated that the chickpea TLP, CaTLP1, participates in osmotic stress response and might be associated with ABA-dependent network. However, how CaTLP1 is connected to ABA signaling remains unclear. The CaTLP1 was found to be engaged in ABA-mediated gene expression and stomatal closure. Complementation of the yeast *yap1* mutant with CaTLP1 revealed its role in ROS scavenging. Furthermore, complementation of *Arabidopsis attlp2* mutant displayed enhanced stress tolerance, indicating the functional conservation of TLPs across the species. The presence of ABA-responsive element along with other motifs in the proximal promoter regions of TLPs firmly established their involvement in stress signalling pathways. The *CaTLP1* promoter driven GUS expression was restricted to the vegetative organs, especially stem and rosette leaves. Global protein expression profiling of wild-type, *attlp2* and complemented *Arabidopsis* plants revealed 95 differentially expressed proteins, presumably involved in maintaining physiological and biological processes under dehydration. Immunoprecipitation assay revealed that protein kinases are most likely to interact with CaTLP1. This study provides the first demonstration that the TLPs act as module for ABA-mediated stomatal closure possibly via interaction with protein kinase.

Plants respond and adapt to water-deficit or dehydration conditions by altering their architecture, cellular metabolism and activating various defense machineries. Such responses to dehydration are regulated by differential expression of many genes/gene-products, which are involved in a number of different but potentially overlapping, signal transduction pathways. Analysing the functions of stress-responsive genes is critical to better understand the molecular mechanism governing stress response, which ultimately opens up new avenues for improving stress tolerance. The past decades have seen the prospect of genetically modified plants and testing their potential to modulate tolerance through alteration of the osmolyte levels and enzymes that scavenge reactive oxygen species (ROS), components involved in signal transduction, transcription factors (TFs) and regulators.

Abscisic acid (ABA) level, under stress conditions, increases dynamically in vegetative organs, triggering adaptive responses that are essential for plant survival and productivity^1^. It induces stomatal closure and minimizes water loss through transpiration. On the contrary, high levels of ABA cease overall plant growth^2^. Numerous genes have been reported that display deregulated expression under stress conditions, and many of them are mediated by ABA^3^,^4^. This expression is modulated by the binding of various TFs onto *cis*-regulatory regions of a gene, which act as molecular switches controlling various biological processes including abiotic stress and hormone responses. The TFs have proven quite useful in improving stress tolerance in transgenic plants, through influencing expression of a number of stress-responsive genes^5^,^6^.

The tubby proteins are encoded by a multigene family. The first tubby gene was identified in mice, and shown to be associated with late-onset obesity and neurosensory deficits^7^. The proteins that are homologous to tubby are known as tubby-like proteins (TLPs). The members of tubby family have been found to perform multiple roles including vesicular trafficking, insulin signalling, gene transcription, G-protein signalling, and ribosomal RNA synthesis^8^, among others. Tubby proteins are present in organisms ranging from unicellular to multicellular eukaryotes^9^. While in mammals, these proteins form a small family, which consists of Tubby, and four TLPs^10^, plants appear to harbor a large number of TLPs^9^,^10^,^11^. This expansion is the result of segmental duplication and random translocation and insertion^12^ indicating a significant role of TLPs in plants. However, exploration of the molecular function of these proteins remains still elusive. Recent studies have explored the role of TLPs in abiotic stress signalling^9^, and patho-stress^11^,^13^. In a previous proteomics study, we identified the chickpea TLP, designated CaTLP1 in the extracellular matrix^14^, and demonstrated that CaTLP1 is a putative transcription factor presumably involved in multivariate stress^15^.

Biological processes are executed by proteins that, to a large extent, depend on interactions with other proteins for their activity. These interactions are specific, even among members of a particular protein family that contain similar interaction domains. Studying these specific interactions reveals the molecular network that may lead to potential functional linkages and molecular explanations of biological processes in an organism. Several previous studies established the significant findings in the study of protein complexes formed by ectopically overexpressed proteins from same as well as other plant species^16^,^17^. To date, generation of large-scale protein-protein interaction (PPI) maps has relied on the yeast two-hybrid system, which detects binary interactions through activation of a reporter gene expression^18^. However, ultrasensitive mass spectrometric identification is capable of directly identifying the protein complexes on a proteome-wide scale^19^. The advantage of analyzing purified protein complexes is the ability to identify specific interacting proteins and post-translational modifications that may otherwise go undetected in large-scale global analyses. This is achieved by performing an immunoprecipitation with a bait protein and analyzing the prey proteins using mass spectrometry^20^. Insight into individual pathways can be obtained by aiming a particular group of proteins that is reported to be enriched for interactions. Interactions of TFs increase the selectivity of protein-DNA interactions and create a large number of diverse DNA-binding complexes from a relatively small number of proteins. In this study, we investigated the potential role of CaTLP1 in modulating plant responses to abiotic stress using mutant, complemented and overexpressing lines of *Arabidopsis*. We showed that the CaTLP1 equips plants to withstand such stress through stomatal modulations and regulation of gene expression via ABA-dependent pathways. Further, we used mass spectrometry based interactive proteomics for the identification of CaTLP1-complex components that mediate stress tolerance.

## Results

### CaTLP1 counteracts oxidative stress in yeast

**Figure 1:**
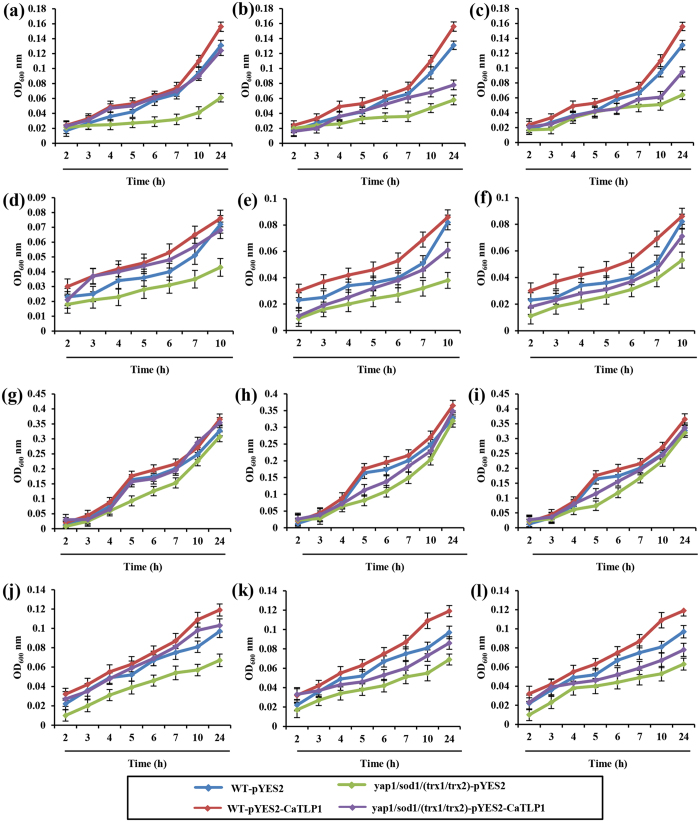
Heterologous complementation of yeast mutants by CaTLP1. Wild-type and *yap1* (**a,d,g,j**) sod1 (**b,e,h,k**), trx1/trx2 (**c,f,i,l**) yeast mutants, transformed with pYES2 and pYES2-CaTLP1, were cultured in SD-URA media overnight and diluted in fresh media (approximate OD_600_ value of 0.01) before being subjected to (**a–c**) 5 mM H_2_O_2_, (**d–f**) 1.2 M NaCl, (**g–i**) 0.1 M LiCl and (**j-l**) 0.5 mM menadione treatments. The cultures were incubated at 30 °C and OD_600_ readings were taken at every 1 h. Data shown are the means ± SD of three biological replicates.

In a previous study, we demonstrated that CaTLP1 could impart improved tolerance against multivariate stresses^15^, possibly through regulation of antioxidant defense genes. Detoxification of ROS is a common mechanism of plant adaptation or tolerance to oxidative stress. The budding yeast, *S. cerevisiae* harbors the same oxidative stress defense mechanisms as in higher eukaryotes^21^ and therefore, to examine the precise role of CaTLP1 in ROS-scavenging, functional complementation was carried out in yeast mutants *viz. yap1, sod1* and *trx1/trx2* affected in antioxidant function. The coding sequence of *CaTLP1* was cloned in expression vector pYES2 and introduced in *yap1, sod1* and *trx1/trx2* in the BY4741 background. The transformants were selected on auxotrophic media and tested for their growth phenotype on YPD media containing various stress-inducing chemicals (0.5 mM menadione, 1.2 M NaCl, 5 mM H_2_O_2_ and 0.1 M LiCl). While there was inhibition of growth under menadione and H_2_O_2_ treatments, the growth phenotype of the mutant in the plate containing other stressors was not very significant from that of WT strains (Supplemental Fig. 1a). However, the OD based observation in liquid media indicated that expression of CaTLP1 could enhance the survival of *yap1* when compared with *sod1* and *trx1/trx2* mutants (Fig. 1). The mutant strains transformed with empty vector could not rescue the growth defect. These results revealed that CaTLP1 could rescue the suppressed activity in the yeast mutants, suggesting its key role in regulation of ROS.

### Expression of CaTLP1 alters stress response in transgenic *Arabidopsis*

To explore *in planta* function of CaTLP1 in stress response, we complemented its homolog in *Arabidopsis*. When searched against TAIR database, *AtTLP2* was found to be the closest ortholog of *CaTLP1* in *Arabidopsis*. T-DNA mutant (SALK_058100 C) was selected as null mutant and complemented lines with CaTLP1 were generated. On an average, 4–6 homozygous lines, both complemented and overexpressing transgenics, were obtained in T4 generation. The wild-type, CaTLP1 overexpressing line (OE-11) and complemented plants (OEC-3, OEC-6) along with *attlp2* mutant were selected for further studies.

**Figure 2:**
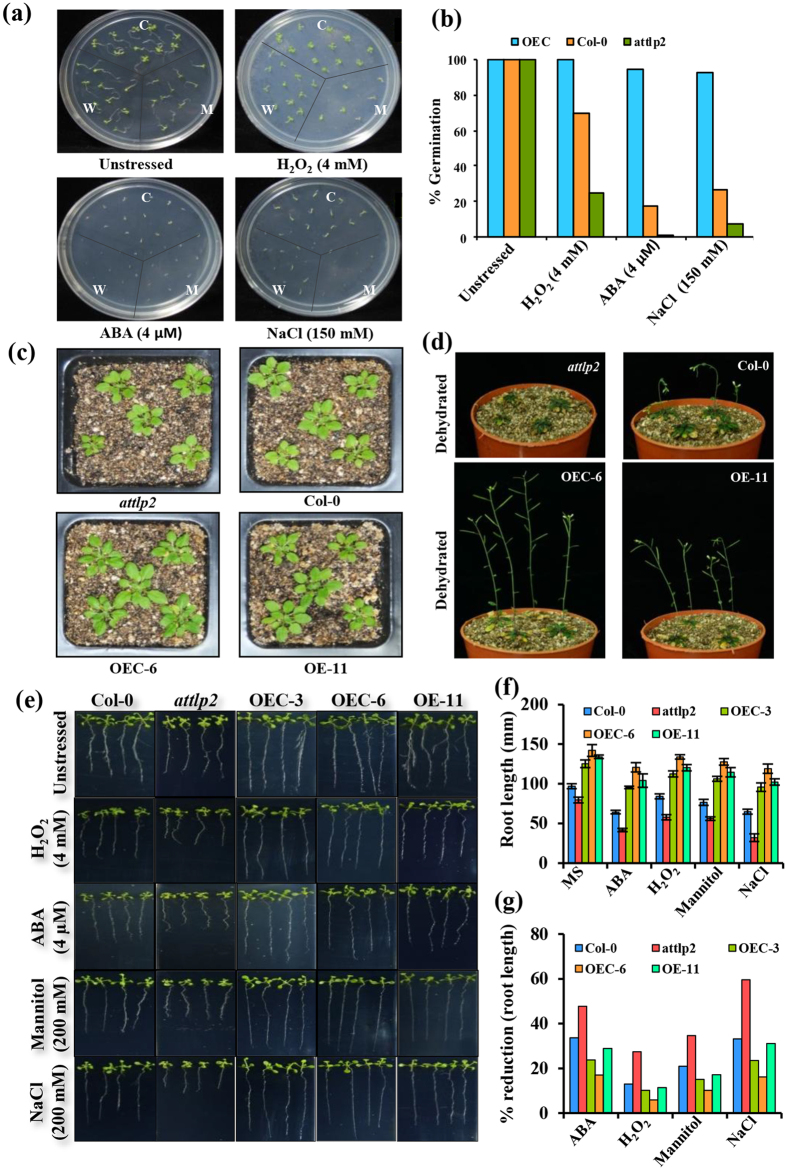
Effect of stress on different stages of wild-type, mutant and transgenic *Arabidopsis*. (**a**) Germination assay of wild-type, *attlp2*, complemented (OEC-3, OEC-6) and overexpressing (OE-11) line(s) on MS media supplemented with H_2_O_2_ (4 mM), ABA (4 μM), and NaCl (200 mM). (**b**) Seed germination percentage under various stresses. (**c**) Morphology of 3-week-old seedling of the indicated lines. (**d**) Morphological features of 4-week-old plants of the indicated lines subjected to dehydration for 10 d. (**e**) Seedlings at four-leaf stage were transferred to various stresses as indicated and growth was monitored after 7 d. (**f**) Histogram showing the differential root length. (**g**) Percent reduction in root growth of the indicated lines.

The transgenic plants were tested for their seed germination and survival under stress conditions. Consistent with our previous results^15^, the overexpressing plants showed better phenotype as compared to mutants and wild-type counterparts (Fig. 2). The mutants did not show any difference in germination rate as compared to wild-type in unstressed control condition. However, under hypersalinity, osmotic and oxidative stress conditions, the germination rate of CaTLP1 complemented plants were much higher than mutants and wild-type (Fig. 2a,b), indicating early response of CaTLP1 in stress signaling. In a separate set of experiment, the four-leaf stage seedlings were transferred to pots and allowed to grow further. The 3-week-old transgenic plants harboring CaTLP1 showed better growth as compared to the mutants (Fig. 2c). When subjected to stress conditions, the mature transgenic plants exhibited enhanced tolerance to dehydration (Fig. 2d). In parallel, a separate set of pots were kept well-watered. Apart from the complemented lines, there were no significant differences in the phenotype of plants under unstressed condition (Supplemental Fig. 1b). However, the reduction in biomass of complemented line under dehydration was significantly lower as compared to wild type and mutant (Supplemental Fig 2 and Supplemental Table 1). The alteration in root length was monitored on MS plates supplemented with various stressors *viz.* NaCl for hypersalinity, H_2_O_2_ for oxidative stress and mannitol for dehydration, besides ABA treatment. The transgenic seedlings showed less inhibitory effects on root growth as compared to wild-type and *attlp2* mutants (Fig. 2e,f and Table 1). When subjected to ABA treatment, the degree of inhibition in *attlp2* mutants and wild-type was 47 and 33%, respectively as against 28% in overexpressing and 17–23% in complemented lines. The reduction in root length under hypersalinity was 59 and 34% in mutant and wild-type, respectively whereas it was 16–28% in transgenics indicating their better adaptability to such conditions (Fig. 2g).

### CaTLP1 expression influences stomatal movements

**Figure 3:**
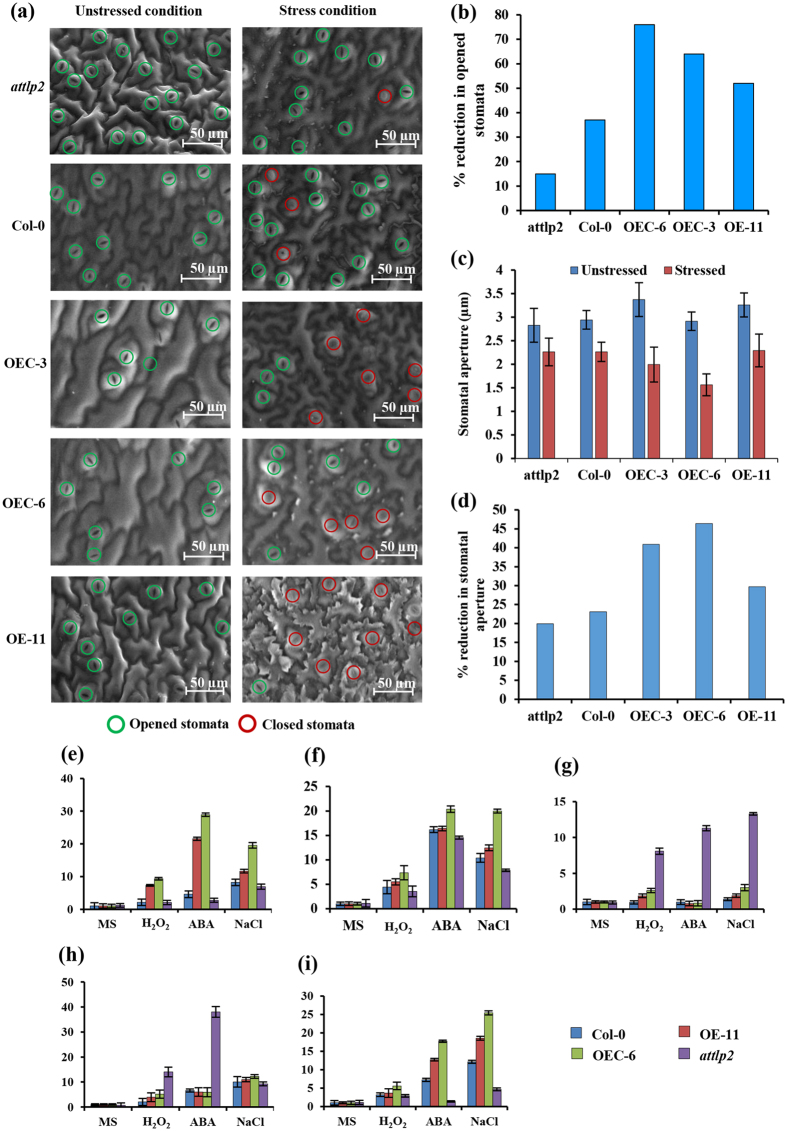
Effect of CaTLP1 on stomatal movement. (**a**) Four-week-old *attlp2*, wild-type and transgenic plants (OEC-3, OEC-6 and OE-11) were uprooted and subjected to dehydration stress. Scanning electron micrographs display the changes of stomatal movement in transgenics with respect to mutant and wild-type plants. The bar indicates the extent of resolution. (**b**) Percentage reduction in number of opened stomata (aperture ≥2 μm). (**c**) Reduction in the stomatal aperture in response to dehydration stress. (**d**) Histogram shows the percent reduction in stomatal aperture for the indicated lines. The results are the means ± SE for 120 stomata. Transcript abundance of stress-responsive genes *viz., RD20* (**e**), *RAB18* (**f**), *bZIP20* (**g**), *polyubiquitin* (**h**) and *GroEL* (**i**) were quantified. Quantitative real time PCR was performed using gene-specific primers (Supplemental Table 5). The relative gene expression was evaluated using comparative cycle threshold method with *EF-1α* as the reference gene. Data shown are the means ± SD of three biological replicates.

Stomatal closure is the most rapid response at the onset of water-deficit condition followed by, on a longer time scale, reduced plant growth and decreasing transpiration thereby facilitating conservation of water^22^. Therefore, we examined the effect of CaTLP1 on stomatal movements. Under unstressed control condition, the stomata in wild-type, mutant and transgenic plants remained open (Fig. 3a). The size of leaf epidermal cells in the transgenics was larger than the mutants and wild-type plants. These results are in consistent with our previous observation where CaTLP1 overexpressing tobacco plants displayed better stress adaptation by maintaining higher photosynthetic rate and reduced water loss^15^. Upon treatment with dehydration, the overexpressing and complemented plants showed inhibition of stomatal opening by 75–80% as against 40–50% in wild-type and 20–30% in mutants (Fig. 3b). Examination of stomatal aperture in leaves under dehydration showed that, relative to wild-type plants, CaTLP1 overexpressing plants had smaller stomatal apertures and *attlp2* mutants had larger stomatal apertures (Fig. 3c,d). These results suggest that the overexpression of CaTLP1 might be involved in stomatal movement, which affect guard cells morphology, and in turn lead to better adaptation.

### Overexpression of CaTLP1 induces stress-responsive genes

The positive effect of CaTLP1 overexpression on abiotic stress tolerance prompted us to check the transcript abundance of stress-responsive marker genes. When subjected to multivariate stress conditions, the expression of *RD20, RAB18, GroEL, polyubiquitin* and *bZIP20* in the transgenic plants was significantly different from that in the mutants and wild-type (Fig. 3e–i). The comparison showed early inductions of *RD20, RAB18,* and *GroEL*, while polyubiquitin expression was delayed in the transgenics. The expression pattern of *GroEL* was found to be opposite to that of *polyubiquitin*. Under the same experimental conditions, the transcripts of *bZIP20* were highly induced in the mutants when compared with wild-type and transgenics. These results altogether suggest a possible role of CaTLP1 in the regulation of expression of downstream stress-responsive genes.

### Expression of CaTLP1 influences proteome profiles

**Figure 4:**
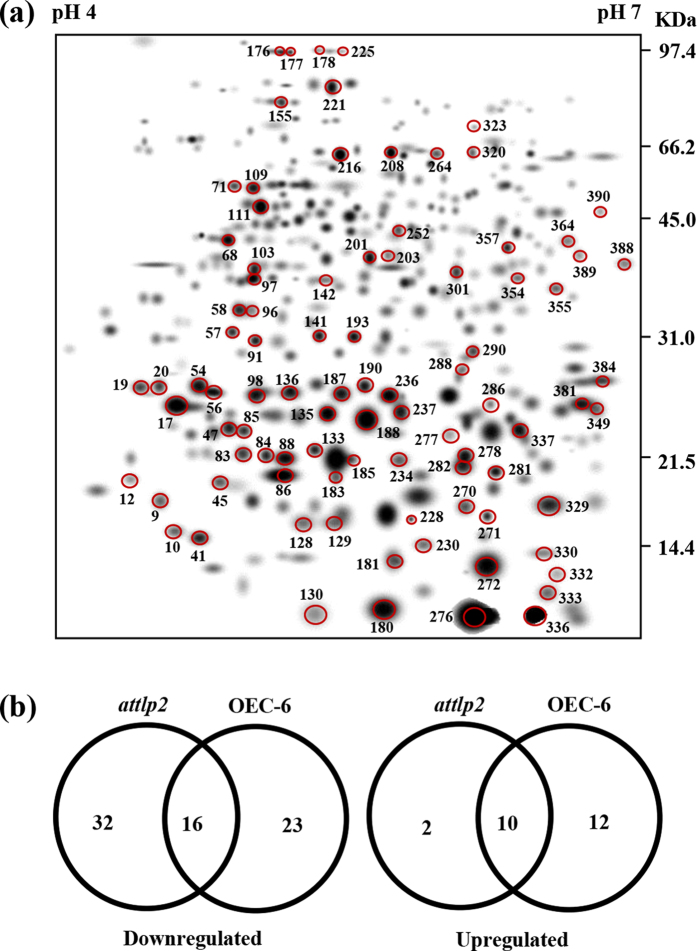
Comparative proteome profiling displaying differentially expressed proteins. (**a**) The higher level matchset was created *in silico* from three standard gels for each of the indicated lines. The numbers were assigned to the spots by PDQuest software (version 7. 2. 0). (**b**) Venn diagram showing the distribution of differentially expressed proteins in *attlp2* and complemented (OEC-6) lines as against wild-type plants.

Next we investigated whether expression of CaTLP1 has any influence on the proteome profiles of wild-type, mutants and complemented plants. Three replicate 2-DE gels of each line were computationally combined into representative standard gels. The proteome of wild-type, mutant and the representative complemented line (OEC-6) displayed 195, 164 and 240 protein spots, respectively. A second level matchset was then created (Fig. 4a). The intensity of spots was normalized to that of landmark protein spots used for internal standardization. The filtered spot quantities were assembled into a data matrix, which consisted of 390 spots, indicating change in intensity for each spot. Approximately 94% of the spots on the standard gels were of high quality, reflecting the reproducibility of the replicates. Quantitative image analysis revealed a total of 95 protein spots that changed their intensities significantly by more than 2.5-fold. The differentially regulated proteins in *attlp2* and OEC-6 are represented by Venn diagram (Fig. 4b).

### CaTLP1 promoter harbors stress-responsive regulatory elements

**Figure 5:**
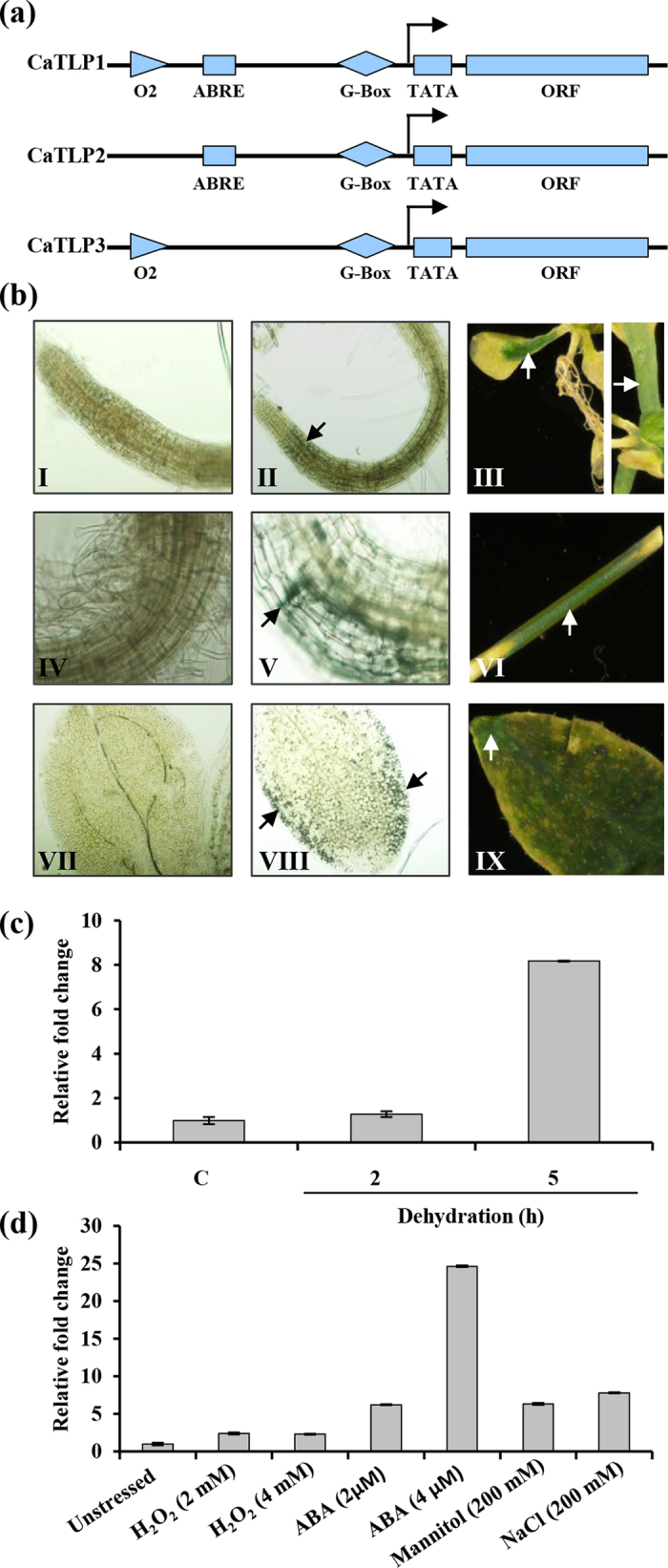
Proximal regulatory elements of *CaTLP1* and influence on ABA-mediated gene expression. (**a**) Comparison of regulatory elements in the proximal promoter region of chickpea TLPs. (**b**) Expression of *ProCaTLP1-GUS* in *Arabidopsis.* GUS expression is shown in root (I), epicotyl (IV) and leaf (VII) of wild-type; root (II), epicotyl (V) and leaf (VIII) of 10-d-old transgenic seedlings; root (III), stem (VI) and leaf (IX) of 6-week-old transgenic plants. Transcript accumulation of *GUS* under progressive dehydration (**c**) and various other stresses (**d**). Data shown are the means ± SD of three biological replicates.

Since CaTLP1 was found to have a regulatory role in stress tolerance, we performed an *in silico* analysis of CaTLP promoter region. A 1.4 kb 5′-upstream region of *CaTLP1* was amplified by genome walking approach. The 5′-upstream regions of two paralogs of CaTLP, designated CaTLP2 and CaTLP3, were retrieved from chickpea draft sequence^23^. Using PLACE and PLANTCARE databases, we analysed the sequences and predicted the key *cis*-acting elements such as ABRE, MYBPLANT, MYCCONSENSUS and ROOTMOTIF, among others (Supplemental Table 2), albeit the number of predicted *cis*-elements differed in the paralogs. The analysis revealed that most elements in *CaTLP* promoters were mainly environment- or hormone-responsive. A putative TATA box motif was found at 198 bp upstream of the *CaTLP1* ORF, while O2-site and G-box sequence was found at 228 bp and 408 bp, respectively. G-box element was found with Box-1 and Box-4 at the upstream of the *CaTLP2* and *CaTLP3* ORFs, respectively. These motifs along with the ABRE form various types of ABRE cassettes, which help in effective regulation by ABRE motif itself. Interestingly, ABRE motif was present in CaTLP1 and CaTLP2, but not in CaTLP3 (Fig. 5a), suggesting the different functions of TLPs in chickpea. We, therefore, propose that *CaTLP1* promoter is an inducible promoter and might be regulated by multiple abiotic stress factors and hormones.

The genomic sequence upstream of the *CaTLP1* orthologues in other plant species were retrieved from the NCBI and Phytozome database (http://www.phytozome.net/) and subjected to comparative analysis by multiple alignment with ClustalW and PlantCARE programs. The analyses revealed the presence of consensus ABRE sequences (Supplemental Table 3) with several common *cis*-acting elements in most orthologs.

### CaTLP1 is predominantly expressed in vegetative organs

To study the tissue-specific expression and transcriptional regulation of *CaTLP1*, we developed *ProCaTLP1:GUS* lines in *Arabidopsis.* Tissue samples from 6-d-old seedlings and 4-week-old plants were analyzed for GUS-staining. The fusion protein showed distinct expression profile across developing stages (Fig. 5b). GUS-staining was detected in roots, leaves and epicotyls of young seedlings, and was significantly higher in mature vegetative organs, especially stem and rosette leaves but not in roots. Interestingly, promoter activity was observed predominantly in leaf blade of matured plants, whereas in seedlings, maximal activity was restricted to the leaf edges. These results indicate overlapping but distinct expression pattern of CaTLP1 during developmental stages.

### ABRE regulon of CaTLP1 is involved in osmotic stress responses

Since ABRE is known as a *cis-*element involved in ABA signaling, we surmised that the ABRE sequence in the *CaTLP1* promoter might be involved in ABA-mediated signaling. The 10-d-old seedling expressing *ProCaTLP1*-*GUS* were grown on MS media supplemented with ABA and other stressors *viz.* H_2_O_2_, mannitol and NaCl in various concentrations for 5 days, and the transcript abundance of *GUS* was quantified. The transcript level increased 2- to 9-fold upon dehydration for 5 h (Fig. 5c), and 3-, 8- and 6-fold with H_2_O_2_, NaCl and mannitol treatments, respectively (Fig. 5d). The accumulation of transcripts was much higher upon treatment with 2 and 4 μM ABA (approximately 9- and 25-fold, respectively). These results indicate that the ABRE sequence might be responsible for the osmotic stress induced expression of *CaTLP1* via ABA-dependent pathway.

### CaTLP1 complexes with kinase family proteins as interacting partners

**Figure 6:**
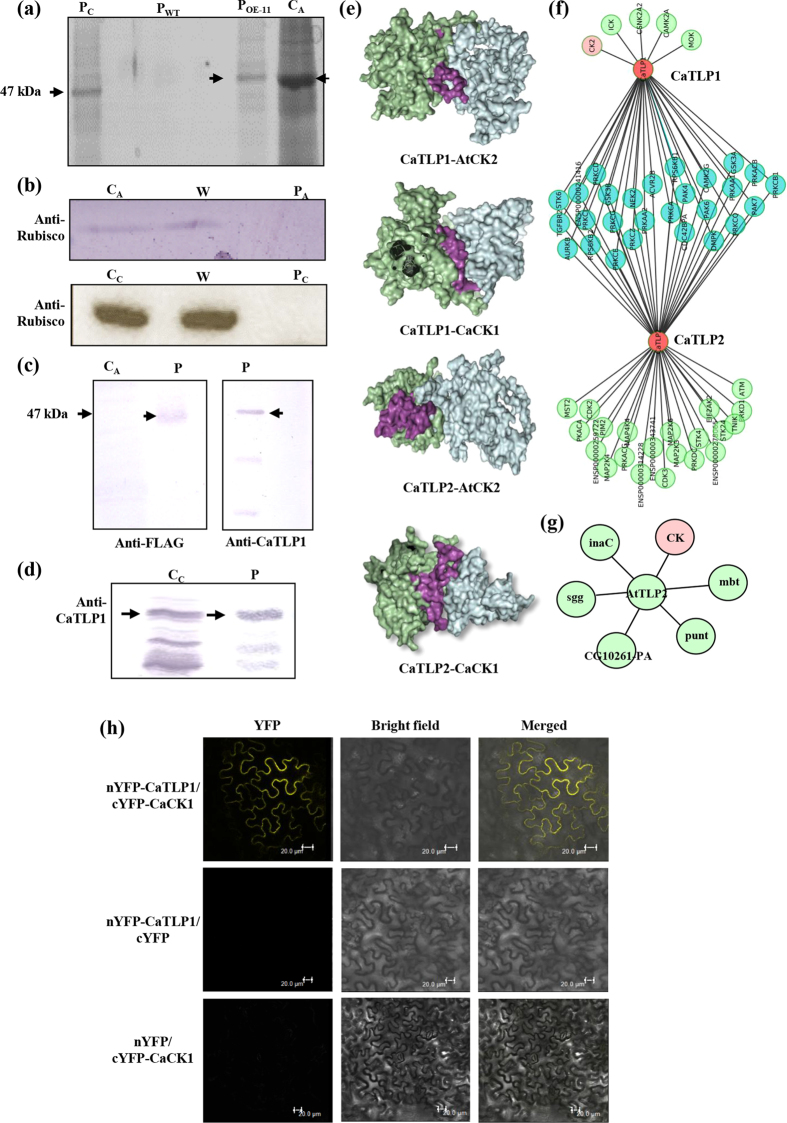
Putative interacting partners of CaTLPs. (**a**) CBB-stained SDS-PAGE gel showing protein bands immunoprecipitated from transgenic *Arabidopsis* (OE-11) and chickpea, probed with anti-FLAG and anti-CaTLP1 antibodies, respectively. (**b**) Western blot analysis of immunoprecipitated proteins with anti-Rubisco antibody. (**c,d**) Confirmation of immunoprecipitates using anti-FLAG and anti-CaTLP1 antibodies. C_C_, crude protein from chickpea; C_A_, crude protein from *Arabidopsis*; P, purified protein; P_A_, purified protein from *Arabidopsis*; P_C_, purified protein from chickpea; W, wash fraction; P_WT_, purified protein from wild-type plants. Arrows indicate band of purified CaTLP1. (**e**) Surface view interaction of F-Box domain (purple) of CaTLP1 and CaTLP2 (green) with casein kinase 1-like protein 2 (AtCK2) and CaCK1 (cyan) visualized with PyMOL. (**f**) Interactions of CaTLP1 and CaTLP2 with kinase family proteins showing common as well as exclusive members in the association network. (**g**) AtTLP2 interaction showing the common casein kinase family protein CK (pink circle) in the network. (**h**) BiFC assay showing interaction of nYFP-CaTLP1 and cYFP-CaCK1.

Our findings demonstrated that CaTLP1 plays a key role in multivariate stress responses. To address how CaTLP1 responds to stress adaptation, we investigated the interactome using transgenics overexpressing CaTLP1-FLAG fusion protein (OE-11). The protein complex from 5-week-old transgenic plants was immunoprecipitated using anti-FLAG antibody. Similarly anti-CaTLP1 antibody was used to immunoprecipitate CaTLP1-protein complex from 4-week-old chickpea plants. The immunoprecipitates were separated by SDS-PAGE, followed by detection with CBB staining (Fig. 6a). The purity of immunoprecipitates was confirmed by anti-Rubisco antibody (Fig. 6b). Protein band corresponding to 47 kDa, from purified fraction, indicated the presence of CaTLP1 which was further confirmed by immunoblot analysis. The immunoprecipitates displayed few extra bands in both chickpea and transgenic *Arabidopsis*, indicating possible interacting proteins. Additionally, immunoblot analyses with anti-FLAG and anti-CaTLP1 antibodies confirmed the presence of CaTLP1 in the immunoprecipitates (Fig. 6c and d).

Next we performed mass spectrometry analysis to identify the proteins present in the immunoprecipitates using LC-ESI/MS/MS. A total of 12 proteins were identified in the immunoprecipitates. A critical screening revealed 10 proteins to be potential interacting partners, most of which were from the protein kinase family (Supplemental Table 4). Casein kinase 1-like protein 2 (CK2) from *Arabidopsis* and protein kinase from chickpea exhibited the highest Mascot score, indicating most likeliness to interact with CaTLP1.

### CaTLP1 and CaTLP2 interact with protein kinases through F-box

Previous studies demonstrated the interaction of casein kinase with proteins harboring F-box domain^24^,^25^. Thus, we performed computational docking experiments to predict the contact interface of CaTLP1 and CaTLP2 with *Arabidopsis* CK2 and chickpea protein kinase (designated CaCK1). The results suggest that CK2 and CaCK1 interact with CaTLPs through F-box domain (Fig. 6e). To further validate these interactions, system biology approaches were applied to construct the phosphorylation oriented protein kinase interactome. The individual interactions of CaTLP1 or CaTLP2 along with common interactions of the CaTLPs are shown in Fig. 6f. Among 55 interactions, 5 were unique to CaTLP1, 22 were unique to CaTLP2, while 26 interactions were shared by both the TLPs. The two categories of interacting partners shown by the protein kinase interactome suggest the interaction diversity between the two paralogs. AtTLP2 was also predicted to interact with casein kinase and protein kinase family (Fig. 6g). To further validate the interaction between CaTLP1 and CaCK1 *in planta*, a bimolecular fluorescence complementation (BiFC) was performed in tobacco leaf epidermal cells. In this assay, putative partners are fused to inactive N-terminal and C-terminal moieties of YFP. Bait and prey association brings YFP fragments together and drives reassembly of the active fluorescent protein, providing a direct readout of the interaction^26^. In the experiment with CaTLP1 + CaCK1, one out of eight combinations gave positive signal *i.e.* YFP fluorescence, in transfected tissues (Fig. 6h). These results reconfirm that CaTLP1 physically interacts with CaCK1 *in vivo*. Although, CaTLP2 was predicted to interact with protein kinase family and other various classes of kinases, no interaction could be predicted with casein kinases. These results indicate specific interactions and thus distinct functions of various TLPs.

## Discussion

Water deficiency in soil interferes with mineral nutrition and water uptake, which leads to accumulation of toxic ions in plants. To reduce these detrimental effects, plants use several strategies, including the regulated expression of specific proteins, which eventually re-establishes proper cellular ion and osmotic homeostasis with other concomitant processes of repair and detoxification^27^. The machinery leading to the expression of dehydration-responsive genes conforms to the general cellular model, with a complex signal transduction cascade that can be divided into the following steps: (*a*) perception of stimulus; (*b*) processing, including amplification and integration of the signal; and (*c*) a response reaction in the form of *de novo* gene expression.

Kamada *et al*.^28^ proposed an attractive model for the stress-induced activation of a transduction pathway by investigating the heat-shock response in yeast, which may provide the conceptual framework for devising experiments in plants. The exposure of yeast cells to low doses of H_2_O_2_ or O^2^-generating drugs switches on within minutes a resistance to toxic doses of these oxidants^29^. The redox-oxidized ROS balance has been compared in a range of yeast mutants. These include the genes affecting antioxidant function at three distinct levels: transcription factors that induce expression of oxidative stress-responsive genes (*Yap1*), defence enzyme encoding superoxide dismutase (*Sod1*), and protein disulfide bond metabolism (*Trx1* and *Trx2*). Mutants deficient in Yap1p show reduced activities of several enzymes with antioxidant activity such as SOD, glucose-6-phosphate dehydrogenase, and glutathione reductase^30^. The adaptive response to H_2_O_2_ is also severely affected by *yap1* mutants, suggesting that Yap1p affects the transcription of genes involved in such response^31^. It is of significance that the transcription of *TRX2* is severely impaired by Yap1 mutation, yet the *TRX2* transcription is induced by H_2_O_2_, indicating that additional factor(s) might be involved in this process^32^. Yap1 controls a large oxidative stress-responsive regulon, which includes most of the oxidant scavenging enzymes Sod1p and Sod2p. Its function can be activated by several chemical stressors including H_2_O_2_ and menadione^33^, and this activation is attributed to a redox stress-imposed nuclear redistribution of the protein. In view of the above, *yap1* mutant was functionally complemented with CaTLP1, which could enhance its survival (Fig. 1) suggesting a putative role of CaTLP1 in ROS regulation.

Among other responses common in many distinct abiotic stresses, is the induction of cellular architectural changes. Mechanistically distinct abiotic stresses can all give rise to alterations in plant morphology. In this study, we observed that the root growth of CaTLP1 transgenics was much better than mutant and wild-type, when subjected to various stresses (Fig. 2). It is generally believed and supported by correlative evidence that a larger root system might be advantageous to cope with water-deficit conditions. This view has been underpinned by a comparison of near-isogenic lines that differed essentially in the size of their root system^34^. In many circumstances, it is the stress sensitivity of the root that limits the productivity^35^. An improved understanding of molecular responses of roots to such abiotic stresses may therefore facilitate the development of crops with increased tolerance.

During dehydration, stomata are induced to close as leaves sense water-deficit, particularly after the leaf water potential drops below certain threshold level. The production of ABA triggers the increase of cytosolic Ca^2+^ concentration in guard cells both via IP3 signal transduction cascade and also, via cADPR. These processes are correlated with a reduction in stomatal aperture^36^. It is well established that ABA promotes water-deficit tolerance in plants through stomatal regulation^37^. In this context, CaTLP1 might function as a positive regulator of ABA-mediated stomatal closure. To activate stress responses, genetic reprogramming is required, and several hundred genes have thus far been identified as stress-responsive^38^,^39^. Among the early induced genes, RD20, a member of caleosin appears to be one of the most highly expressed and is often used as a stress marker^40^. *CaTLP1* could modulate the expression of several of the marker genes, suggesting that it might play a regulatory role for downstream stress signaling possibly by controlling guard cell morphology (Fig. 3).

Comparative proteomics has previously been used to decipher the differential protein expression in genetically modified plants^41^,^42^. In our investigation, transgenic *Arabidopsis* (OEC-6) and their isogenic controls (*attlp2* mutant and wild-type) were grown side-by-side in environmentally controlled conditions. By cross-comparing the proteome profiles of these lines, we were able to eliminate the natural variability as well as the environmental effects and determine the possible effects related to differential gene expression as a consequence of transgene expression. Previous studies suggested that the differences in spot quantity between transgenic and non-transgenic lines fell in the range of natural variation or were part of the intended effects^43^,^44^. However, the fact that quite a high number of protein spots were found to be significantly modulated in CaTLP1 transgenics (Fig. 4) is in consistence with the results found in the previous proteomics studies of transgenic maize^41^ and potato^45^.

The promoter sequence of TLPs harbors multiple stress-responsive *cis*-acting elements (Fig. 5). It is interesting that the *cis*-acting elements were nearly identical, despite their low homology. The comparison of the *CaTLP1* promoter with its orthologs in other eudicot species revealed a conserved ABRE motif, which might have a crucial role in its function. The regulation of ABA- and/or stress-responsive gene expression by ABRE and their putative cognate *trans*-acting factors have been studied extensively^46^,^47^. The *CaTLP1* promoter contains other *cis-*elements such as O2-site and G-box besides ABRE (Supplemental Table 2), which are known to be involved in ABA-induced gene expression, and implicated in stress response. Differential expression profiles of GUS under *CaTLP1* promoter indicated that there exists differential transcriptional regulation of *CaTLP1* across the developmental stages and in response to environmental conditions (Fig. 5). Furthermore, several homologous regulatory motifs were identified which could be recognised by MYB, MYC or ABRE binding factors. It is thus conjectured that *CaTLP1* has specific regulatory system similar to the genes having ABA inducible promoters, which might play a key role in the regulation of expression of *CaTLP1* promoter.

Protein interactions that are clustered based on similar interaction patterns can serve as backbones for more complex molecular networks responsible for a particular function or pathway^16^,^17^. We identified a number of CaTLP1 interacting partners that include *Arabidopsis* CK2 and chickpea protein kinase CaCK1 (Supplemental Table 4). Numerous kinases and phosphatases are known to form distinguishable complexes often with regulatory factors that serve to localize or control activity^48^. The available data indicate that ABA or abiotic stresses induce several TFs, for example, ABF/AREB by triggering their phosphorylation^49^. This phosphorylation of TFs is necessary to induce downstream genes and could occur on the casein kinase II (CKII) phosphorylation sites present in the conserved domains^50^. Lai *et al*.^9^ demonstrated the physical interaction between AtTLP9 and ASK1 (*Arabidopsis* Skp1 like 1) through F-box, which may participate in the ABA signalling pathway during seed germination and early development. It is likely that CaTLP1 harboring F-box might play key role in stress tolerance through reprogramming of downstream stress-responsive genes.

**Figure 7:**
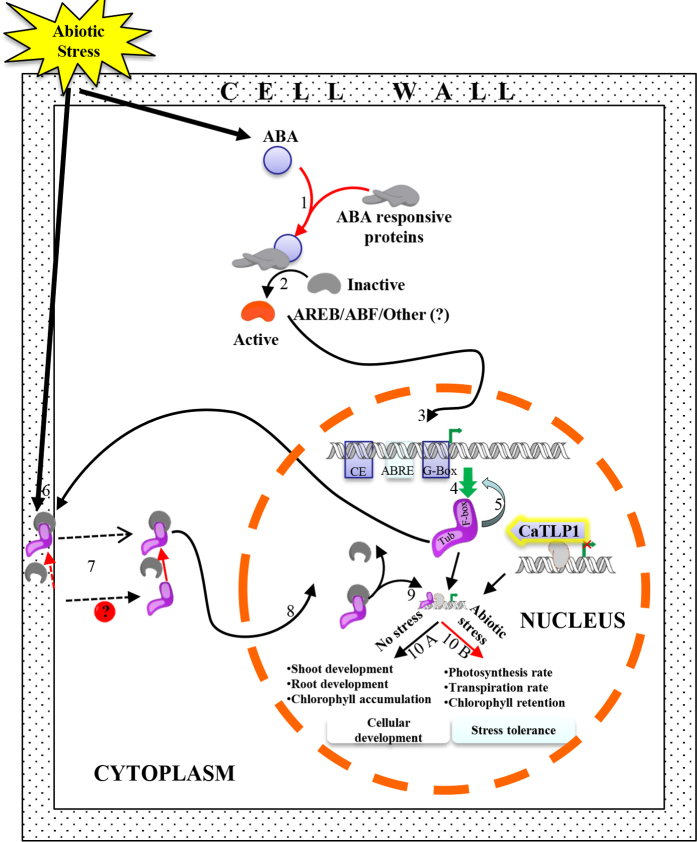
Hypothetical model depicting the possible mechanism of action of CaTLP1. Stress mediated ABA-dependent expression of CaTLP1 (1-4); feed-back inhibition of CaTLP1 (5), perception of signal by CaTLP1 (6); interaction of CaTLP1 with CaCK1 in either possible way (7), nuclear translocation (8); interaction with the upstream regulator (9); and switching on and off the downstream genes (10A and 10B) as detailed in the text. TUB, Tub domain; F-box, F-box domain; R, regulator.

We propose that CaTLP1 may be associated with synaptic function in stress tolerance (Fig. 7). The presence of ABRE in *CaTLP1* promoter suggests the regulation of its expression in an ABA-dependent manner. CaTLP1 comprises two characteristics domains, Tub and F-box and is localized in the extracellular matrix, presumably serving as a molecular sensor for various stress signals. Upon perceiving such signals, it translocates to the nucleus. The translocation might be facilitated by its interaction with CaCK1 protein since casein kinase 2 family proteins are known to translocate extracellular signal-regulated proteins to the nucleus^51^. After being translocated to the nucleus, CaTLP1 might bind to DNA and interact with the regulator bound upstream to the target genes with the help of Tub and F-box domains, respectively. F-box domain either may regulate the activity or recruit other regulators at the promoter that can effectively switch on and off downstream gene expression. The mode of action of these genes is currently unknown, but they might contribute to decreased transpiration through effective stomatal closure and increased rate of photosynthesis under stress conditions. However, under unstressed conditions, these genes render support in cellular development by means of their effect on root and shoot development. Our future efforts would focus on exploring precise mode of functioning and downstream target genes of CaTLP1 in stress response, and to evaluate its use as modifier of plant development and stress tolerance.

## Methods

### Yeast complementation assay

The yeast expression vectors were constructed and transformation was carried out as described previously^52^. The *CaTLP1* coding region was cloned in pYES2 vector (Invitrogen) using BamH1/Not1 restriction sites. The wild-type BY4741 (MATa; his3Δ 1; leu2Δ 0; met15Δ 0; ura3Δ 0) and the mutants *yap1* (MATa; his3Δ 1; leu2Δ 0; met15Δ 0; ura3Δ 0 YML007w::kanMax4)*, sod1* (MATa; his3Δ 1; leu2Δ 0; met15Δ 0; ura3Δ 0 YJR104c::kanMax4) and *trx1*/*trx2 (*MATa; his3Δ 1; leu2Δ 0; met15Δ 0; ura3Δ 0 YGR209c::kanMax4) were transformed with pYES2:CaTLP1 or the empty vector pYES2. Growth assays were performed by inoculating wild-type and transformed strains in the respective medium supplemented with 0.5 mM menadione, 1.2 M NaCl, 5 mM H_2_O_2_ and 0.1 M LiCl. The overnight cultures were diluted to OD_600_ of 0.01 in the respective medium, and growth was monitored at every 1 h at 30 °C.

### Plasmid construction and transformation

The overexpressing CaTLP1 binary vectors were constructed using the full-length cDNA of *CaTLP1.* The cDNA as well as 1.4 kb 5′ upstream sequence were cloned independently into pENTR/D-TOPO vector (Invitrogen). The cDNA was subcloned into the Gateway destination vectors, pGWB411. The *CaTLP1* promoter was cloned into destination vector pGWB433 containing C-terminal *GUS* reporter gene. The gateway cloning was performed using LR Clonase (Invitrogen). The expression plasmids were transformed into *Agrobacterium* strain GV3101.

### Plant materials and growth conditions

Seeds of wild-type (Col-0) and T-DNA mutants were obtained from The *Arabidopsis* Information Resource (TAIR), Carnegie Institution for Science, Department of Plant Biology, Stanford, USA. The seedlings were grown on Pro-Mix soil (Premier Horticulture) in a growth chamber under ambient humidity^53^. Plants were illuminated by Vita-lite fluorescent lamps (Durotest, Fairfield NJ) between 150 and 200 μmol m^−2^ s^−1^ of PAR at 16 h photoperiod. The plants were transformed by the floral-dip method, as previously described^54^ and homozygous seeds were generated for transgenic lines.

### Stress and ABA treatment

Germination assay was performed using approximately 200 surface-sterilized seeds for each line, plated on MS medium without or supplemented with H_2_O_2_ (4 mM), NaCl (150 mM), and ABA (4 μM) and kept at 21 °C with 16 h photoperiod, and rate of germinated seeds were scored daily. Four-leaf staged seedlings (principal growth stage: 1.02–1.03) were grown on MS plates supplemented with H_2_O_2_ (2 and 4 mM), mannitol (200 mM), NaCl (200 mM), and ABA (2 and 4 μM). The seedlings were incubated for 7 d and phenotypic changes were recorded. Primary root length was measured on scanned images using ImageJ software (http://rsbweb.nih.gov/ij). In a separate experiment, 3-week-old plants grown in pots were subjected to dehydration by withdrawing water. Samples were collected and stored at −80 °C unless described otherwise.

### Nucleic acid isolation and analysis

Genomic DNA was isolated using phenol extraction method (http://www.biotech.wisc.edu/ NewServicesandResearch/*Arabidopsis*/). The fragment flanking the T-DNA insert was amplified by PCR using appropriate primers (Supplemental Table 5). RNA was extracted using the TriPURE reagent (Molecular Research Center) and reverse transcribed into cDNA using a poly (dT) reverse primer and Superscript II reverse transcriptase (Invitrogen) after DNase treatment (Roche Diagnostics). The qRT-PCR assays were performed with the ABI PRISM 7700 sequence detection system (Applied Biosystems) using SYBR Green PCR Master mix including cDNA template and appropriate primers (Supplemental Table 5). The internal standard EF-*1α* was used for normalizing the qRT-PCR data and quantification was conducted as described previously^55^.

### Protein extraction and 2-DE

Total proteins were extracted from 4-week-old plants as described earlier^56^. The protein extracts were dissolved in 200 μL rehydration buffer containing 2% immobilized pH gradient (IPG) buffer (pH 4–7), and quantified using 2-D Quant kit (GE Healthcare).

Isoelectric focusing (IEF) was performed with 250 μg proteins per IPG strip (13 cm, pH 4–7) using IPGphor system (GE Healthcare) as described^57^. The strips were then placed on top of 12.5% SDS-PAGE for second-dimension separation at a constant voltage of 100 V. The electrophoresed proteins were visualized by Silver Stain Plus (Bio-Rad).

### Acquisition of image and data analysis

The gel images were scanned using the Fluor-S MultiImager system and analyzed with PDQuest version 7.2.0 (Bio-Rad). To compare spots across gels, a standard image of three biological replicates was created for each sample. The quantity and quality scores to each spot were assigned as described^56^. The correlation coefficient, representing the association between the spot intensities on replicates, was maintained at a minimum of 0.8. The spot densities on the “standard gel” were normalized against the total density in the gel image. To facilitate the comparison of the “standard gels”, the spot volumes were further normalized using three unaltered protein spots across all of the gels. The filtered spot quantities from the “standard gels” were assembled into a data matrix for further analysis.

### Analysis of the 5′-upstream region of CaTLPs

Genome-walking PCR was performed using the Genome Walker kit (Clontech Laboratories). Primary and secondary PCR were conducted using adaptor primers, and adaptor and nested primers complementary to the *CaTLP1* upstream sequence (Supplemental Table 5). The amplicon was cloned into pENTR/D-TOPO vector (Invitrogen). The promoter region of *CaTLP2* and *CaTLP3* were retrieved from the Next Generation Challenge Programme on Chickpea (NGCPC) facility at National Institute of Plant Genome Research, New Delhi, India. Sequence analysis was done using PLACE (http://www.dna.affrc.go.jp/htdocs/PLACE/signalscan.html) and PlantCARE (http://bioinformatics.psb.gent.be/webtools/plantcare/html/) databases.

### Histochemical detection of GUS

GUS histochemical analysis was performed^58^ with tissues or whole seedlings submerged in GUS staining buffer [2 mM 5-bromo-4-chloro-3-indolyl glucuronide, 0.1 M sodium phosphate (pH 7.0), 0.1% (v/v) Triton X-100, 1 mM potassium ferricyanide, 1 mM potassium ferrocyanide and 10 mM EDTA], vacuum infiltrated for 10 min and incubated overnight at 37 °C. The samples were treated with 95% (v/v) ethanol and observed with a stereomicroscope (SZX-ILLD2-200, Olympus).

### Immunoprecipitation

Immunoprecipitation was performed with rProtein A Sepharose Fast Flow (GE Healthcare). Protein extracts were prepared by homogenization of 0.5 g tissue each from wild-type and CaTLP1-FLAG overexpressing *Arabidopsis* lines in 2 mL buffer containing 50 mM HEPES-KOH, pH 7.5, 0.15 M NaCl, 0.5% (v/v) Triton X-100, and 0.1% (v/v) Tween 20. The homogenates were centrifuged at 10,000 g for 15 min at 4 °C to remove cellular debris. The supernatants were incubated with sepharose beads at 4 °C for 16 h to remove non-specific proteins. Beads were separately incubated with anti-FLAG antibody (Agrisera) at 23 ± 2 °C for 1 h. The pre-extracts were then incubated with anti-FLAG conjugated beads at 4 °C for 16 h, and the beads with immunoaffinity complexes were retrieved. The immunoaffinity complexes were eluted with 50 mL of 2x SDS-sample buffer containing 100 mM Tris-HCl, pH 6.8, 4% (w/v) SDS, 20% (w/v) glycerol and 5% (v/v) 2-mercaptoethanol.

### Immunoblot analysis

Immunoblot analysis was performed by resolving proteins on 12.5% SDS-PAGE and electrotransferring onto nitrocellulose membrane at 150 mA for 2 h. The membranes were blocked with 5% (w/v) non-fat milk for 1 h, and incubated with appropriate antibodies for 2 h. The blots were then incubated with alkaline phosphatase conjugated secondary antibody for 1 h and the signals were detected using NBT/BCIP (nitro blue tetrazolium/5-bromo-4-chloro-3-indolyl phosphate) method.

### Mass spectrometry analysis

The immunoprecipitated proteins were subjected to trypsinolysis as described^56^. The peptides were analyzed using a QSTAR Elite mass spectrophotometer (Applied Biosystem) coupled with an on-line Tempo nano-MDLC system. Proteins were identified by the Mascot search algorithm^56^. The function of proteins was assigned using a protein function database Pfam (http://www.sanger.ac.uk/software/Pfam/) or Inter-Pro (http://www.ebi.ac. uk/interpro/).

### *In silico* protein interaction analysis

The interactions of CaTLP1, CaTLP2, casein kinase 1 like 2 and CaCK1 were carried out in the global network using homology modeling. Each of the candidate proteins was searched for their corresponding template via PDB-BLAST. Interaction of CaTLP1 and CaTLP2 was performed with proteins identified in immunoprecipitated complexes from *Arabidopsis* and chickpea using standalone ZDOCK version 3.0.2. NetPhosK 1.0 server (http://www.cbs.dtu.dk/services/NetPhosK/) was used to predict the probable sites of phosphorylation and the kinase-specific interactome was constructed in NetworKIN (version 2.0)^59^. Visualization and analysis of interaction networks were accomplished on Cytoscape version 2.8.1.

### BiFC assay

Coding sequences were cloned in fusion with the N- and C-terminal parts of YFP under the control of the *CaMV35S* promoter in the BiFC vectors. The fluorescence complementation was assayed by transient expression using all eight compatible combinations between protein pairs. Each expression vector was introduced in *Agrobacterium tumefaciens* strain GV 3101 by electroporation. The bacterial cultures were incubated overnight at 28 °C. Each culture was pelleted, washed, and resuspended in infiltration buffer [for 50 ml buffer 250 mg D-glucose, 5 ml of 500 mM MES, 5 ml of 20 mM Na_3_PO_4_.12H_2_O 5 μl of 1 M acetosyringone] to an OD_600_ of 0.5. The inoculum was delivered to the lamina tissue of *N. benthamiana* leaves by infiltration through the lower epidermis. To enhance transient expression of BiFC fusion proteins, the P19 viral suppressor of gene silencing was coexpressed^60^. YFP fluorescence was detected 2 to 3 d after infiltration.

### Statistical analysis

All the experiments were performed in triplicate. The experimental results were expressed as the mean ± standard deviation (SD) using SPSS (Statistical Program for Social Sciences, SPSS Corporation, Chicago, Illinois, USA). Further, one-way analysis of variance (ANOVA) and post hoc analysis with 2-sided Tukey’s HSD were performed at p ≤ 0. 05.

## Additional Information

**How to cite this article**: Wardhan, V. *et al*. Chickpea transcription factor CaTLP1 interacts with protein kinases, modulates ROS accumulation and promotes ABA-mediated stomatal closure. *Sci. Rep.* **6**, 38121; doi: 10.1038/srep38121 (2016).

**Publisher's note:** Springer Nature remains neutral with regard to jurisdictional claims in published maps and institutional affiliations.

## Supplementary Material

Supporting Information

## Figures and Tables

**Table 1:** Effect of abiotic stress on root-growth of *Arabidopsis* seedlings of the indicated lines.

Lines	Treatment				
	MS	ABA	H_2_O_2_	Mannitol	NaCl
Col-0	96.8938 ± 3.08^b^	64.2735 ± 2.28^b^	84.3000 ± 3.14^b^	76.6393 ± 3.85^b^	64.7663 ± 3.16^b^
*attlp2*	79.6433 ± 3.59^a^	41.6223 ± 2.09^a^	57.7862 ± 3.24^a^	56.1120 ± 2.33^a^	32.1800 ± 4.83^a^
OEC-3	125.1643 ± 4.80^c^	95.4465 ± 1.50^c^	112.4735 ± 3.72^c^	106.2707 ± 3.22^c^	95.7710 ± 5.26^c^
OEC-6	142.0960 ± 7.38^d^	120.7598 ± 5.82^d^	133.8245 ± 2.76^e^	127.6842 ± 4.01^d^	119.1500 ± 5.81^d^
OE-11	134.0040 ± 2.26 ^cd^	104.0530 ± 8.34^c^	120.3580 ± 3.97^d^	114.4997 ± 5.91^c^	102.2810 ± 4.11^c^

The superscript letters indicate significantly different at p < 0.05, as measured by 2-sided Tukey’s HSD between the indicated lines. Values represented as the mean ± SD.
