# Supplementary material for: Chickpea transcription factor CaTLP1 interacts with protein kinases, modulates ROS accumulation and promotes ABA-mediated stomatal closure
Source: Sci Rep. 2016 Dec 9;6:38121. doi: 10.1038/srep38121 (PMC5146945; doi:10.1038/srep38121)
Supplement: Supporting Information [file srep38121-s1.pdf]

Chickpea transcription factor CaTLP1 interacts with protein kinases, modulates ROS accumulation and promotes ABA-mediated stomatal closure

Vijay Wardhan, Aarti Pandey, Subhra Chakraborty and Niranjan Chakraborty§

National Institute of Plant Genome Research, Jawaharlal Nehru University Campus, Aruna Asaf Ali Marg, New Delhi-110067, India.

Running title: *CaTLP1 promotes ABA-mediated stomatal closure*

Corresponding author

*****Dr. Niranjan Chakraborty

National Institute for Plant Genome Research

Jawaharlal Nehru University Campus

Aruna Asaf Ali Marg, New Delhi-110067, India.

E-mail: nchakraborty@nipgr.ac.in

Tel: 00-91-11-26735178

Fax: 00-91-11-26741658

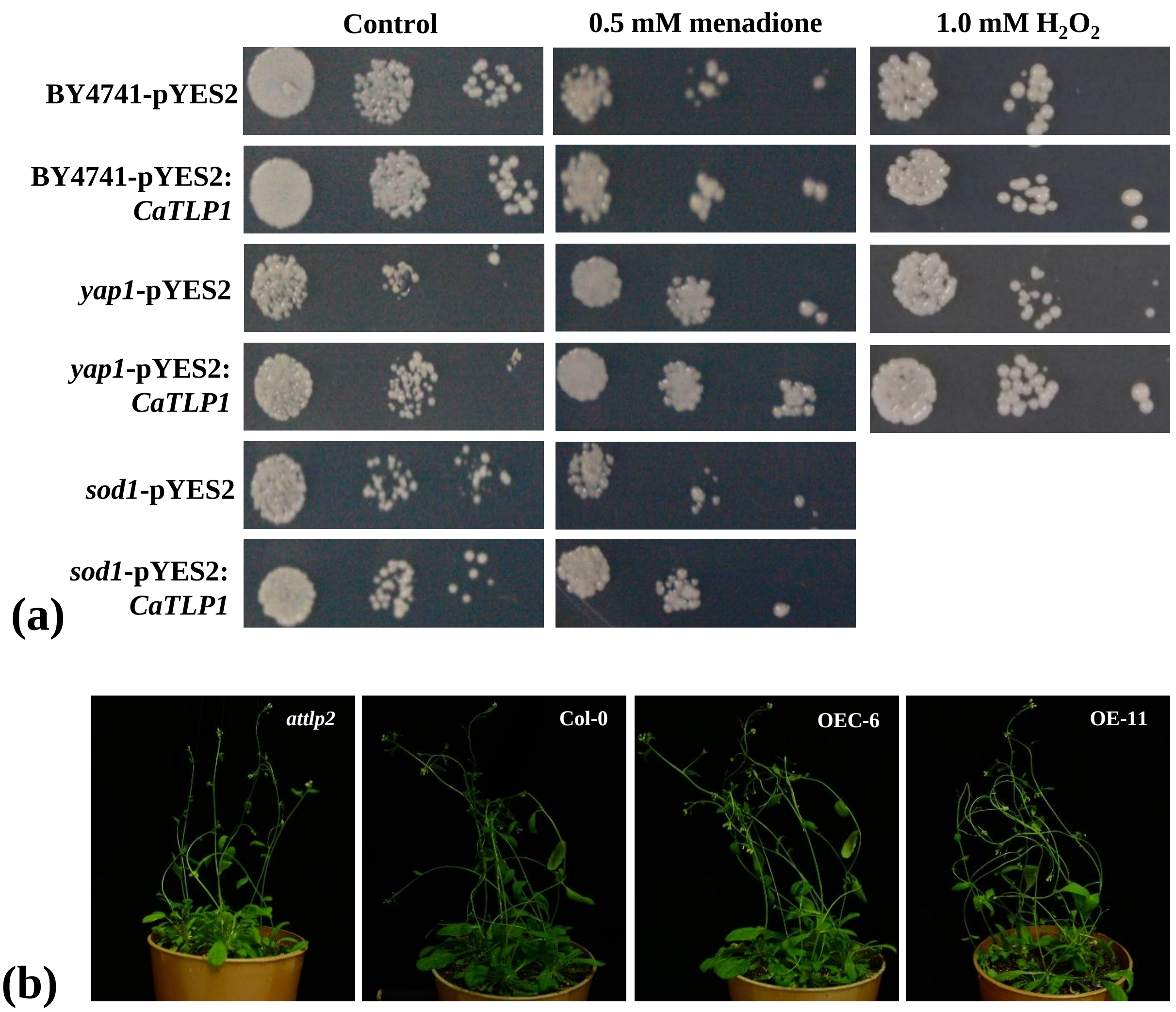

Supplemental Figure 1: (a) Growth phenotype of yeast strains subjected to 0.5 mM menadione and 1.0 mM H_2O_2. Growth was monitored by optical density at 600 nm and cells were taken as 1:10 serial dilution. (b) Growth phenotype of Arabidopsis plants *attlp2*, wild-type (Col0), complemented (OEC-6) and overexpressed (OE-11) in unstressed condition.

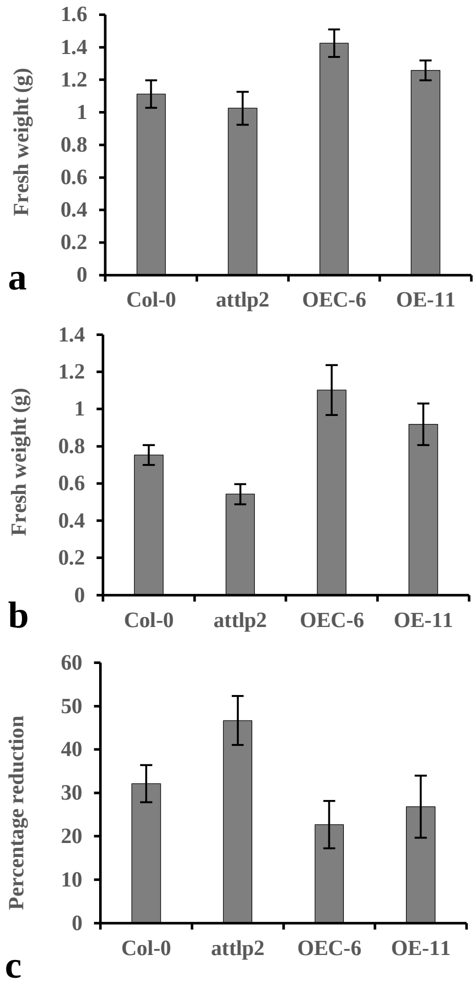

Supplemental Figure 2. Effect of dehydration on growth and development of *Arabidopsis* plants. Biomass accumulation (FW) in six weeks old WT (Col0), mutant (*attlp2*) and transgenic (OEC-6 and OE-11) *Arabidopsis* grown under (a) unstressed condition and (b) under dehydration. (c) Percentage reduction in biomass in response to dehydration stress. The seedlings were grown for three weeks before subjecting them to dehydration. The values under fresh weight represent mean of at least twelve individual plants of each line and error bar represents standard error based on three experiments ($p = 0.01$).

Supplemental Table 1. Biomass [FW (in g)] of Arabidopsis seedlings under dehydration.

Lines	Unstressed	Stressed
Col0	$1.1125 \pm 0.08421^{a,b}$	$0.7525 \pm .05315^{b}$
attlp2	1.0250 ± 0.10083^{a}	$0.5425 \pm .05377^{a}$
OEC-6	1.4250 ± 0.08544^{c}	$1.1025 \pm .13401^{c}$
OE-11	$1.2575 \pm 0.06076^{b,c}$	$0.9175 \pm .11177^{b,c}$

The superscript letters indicate significantly different at $p < 0.05$, as measured by 2-sided Tukey's HSD between the indicated lines. Values represented as mean ± SD.

Supplemental Table 2. List of *cis*-regulatory elements present in the proximal promoter region of chickpea TLPs.

I. PLACE database result of ~1.4 kb promoter fragment of each *TLP* is provided.

***cis*-elements**	**CaTLP1**	**CaTLP2**	**CaTLP3**
-10PEHVPSBD	1	2	1
-300CORE	1	0	1
-300ELEMENT	2	3	2
AACACOREOSGLUB1	1	0	1
ABRE3HVA1	0	1	0
ABRELATERD1	1	3	2
ABRERATCAL	0	1	1
ACGTABREMOTIFA2OSEM	1	1	1
ACGTATERD1	2	8	4
AGMOTIFNTMYB2	1	1	1
AMYBOX1	3	2	2
ANAERO1CONSENSUS	1	3	1
ANAERO2CONSENSUS	1	0	1
ANAERO3CONSENSUS	0	1	0
ANAERO5CONSENSUS	0	0	1
ARFAT	2	0	2
ARR1AT	23	30	16
BIHD1OS	1	4	2
BOXIINTPATPB	1	1	1
BOXIIPCCHS	1	0	1
BP5OSWX	0	2	1
BS1EGCCR	0	0	1
CAATBOX1	17	22	18
CACTFTPPCA1	21	14	20
CANBNNAPA	0	1	0
CAREOSREP1	1	2	1
CARGCW8GAT	0	2	0
CBFHV	0	0	2
CCA1ATLHCB1	0	1	0
CCAATBOX1	3	4	5
CGCGBOXAT	0	0	4
CIACADIANLELHC	2	1	2
CPBCSPOR	2	4	1
CURECORECR	2	6	2
DOFCOREZM	19	23	18
DPBFCOREDCDC3	0	2	0
DRE2COREZMRAB17	0	0	2
DRECRTCOREAT	0	0	2
E2FCONSENSUS	0	1	0
EBOXBNNAPA	8	6	8

EECCRCAH1	1	4	5
ELRECOREPCRP1	0	0	1
EMHVCHORD	1	0	1
ERELEE4	2	1	0
GADOWNAT	0	1	0
GARE1OSREP1	1	0	1
GAREAT	2	2	1
GATABOX	6	12	7
GT1CONSENSUS	10	12	15
GT1CORE	2	1	2
GT1GMSCAM4	4	4	5
GTGANTG10	10	9	13
HDZIP2ATATHB2	1	0	1
IBOXCORE	1	3	2
INRNTPSADB	6	5	6
LECPLEACS2	1	0	0
LTRE1HVBLT49	1	0	1
LTREATLTI78	0	0	1
LTRECOREATCOR15	0	0	2
MARTBOX	3	4	3
MYB1AT	2	2	2
MYB1LEPR	1	0	1
MYB2CONSENSUSAT	2	0	1
MYBCORE	5	0	7
MYBCOREATCYCB1	3	1	2
MYBGAHV	2	2	1
MYBPLANT	1	0	2
MYBPZM	1	0	1
MYBST1	1	2	1
MYCATERD1	2	2	1
MYCATRD22	2	2	1
MYCCONSENSUSAT	8	6	8
NAPINMOTIFBN	0	1	0
NODCON1GM	0	1	0
NODCON2GM	4	3	4
NTBBF1ARROLB	2	2	3
OSE1ROOTNODULE	0	1	0
OSE2ROOTNODULE	4	3	4
POLASIG1	12	12	12
POLASIG2	6	2	3
POLASIG3	8	5	8
POLLEN1LELAT52	7	10	6
PREATPRODH	2	0	1
PRECONSCRHSP70A	0	1	0
PROLAMINBOXOSGLUB1	0	1	0
PYRIMIDINEBOXOSRAMY1A	0	3	0

QARBNEXTA	0	1	0
RAV1AAT	7	4	9
RAV1BAT	0	0	1
RBCSCONSENSUS	1	1	2
REALPHALGLHCB21	2	4	2
RHERPATEXPA7	0	3	1
ROOTMOTIFTAPOX1	11	12	1
RYREPEATBNNAPA	1	1	1
RYREPEATLEGUMINBOX	0	1	0
S1FBOXSORPS1L21	1	2	1
SEBFCONSSTPR10A	1	0	2
SEF3MOTIFGM	1	1	1
SEF4MOTIFGM7S	7	7	4
SITEIIATCYTC	1	0	1
SORLIP1AT	3	0	2
SORLIP2AT	3	0	2
SORLIP5AT	0	0	1
SP8BFIBSP8BIB	1	0	0
SREATMSD	0	1	0
SURECOREATSULTR11	3	1	2
SV40COREENHAN	0	1	0
T/GBOXATPIN2	0	2	1
TAAAGSTKST1	6	3	6
TATABOX2	0	1	0
TATABOX3	1	0	0
TATABOX4	1	2	0
TATABOX5	12	8	10
TATAPVTRNALEU	0	1	0
TATCCACHVAL21	0	1	0
TATCCAOSAMY	1	1	1
TATCCAYMOTIFOSRAMY3D	0	1	0
TBOXATGAPB	0	1	1
TCA1MOTIF	0	1	0
WBBOXPCWRKY1	0	0	1
WBOXATNPR1	1	3	2
WBOXHVISO1	2	1	3
WBOXNTCHN48	1	0	1
WBOXNTERF3	3	2	6
WRKY71OS	4	6	8

II. PLANTCARE database result of ~1.4 kb promoter fragment of chickpea TLPs.

***cis*-elements**	**CaTLP1**	**CaTLP2**	**CaTLP3**
AAGAA-motif	√	√	√
ABRE	√	√	x
AE-box	√	x	√

ARE	√	√	√
ATCT-motif	√	√	x
Box 4	√	√	√
Box I	√	√	x
CAAT-box	√	√	√
CAT-box	√	x	x
CATT-motif	√	√	√
EIRE	√	x	√
ERE	√	x	x
F-box	√	x	x
G-box	√	√	√
GATA-motif	√	x	x
GCN4_motif	√	√	√
GT1-motif	√	√	√
LTR	√	x	√
O2-site	√	x	√
Skn-1_motif	√	√	x
TATA-box	√	√	√
TC-rich repeats	√	√	√
TCA-element	√	x	√
TCT-motif	√	x	√
Unnamed__1	√	x	x
Unnamed__3	√	x	x
Unnamed__4	√	√	√
chs•CMA1a	√	x	x
circadian	√	√	x
AAAC-motif	x	√	x
ACE	x	√	x
GAG-motif	x	√	√
GARE-motif	x	√	x
I-box	x	√	x
MRE	x	√	√
P-box	x	√	x
TCCC-motif	x	√	x
as-2-box	x	√	x
Box-W1	x	x	√
MBS	x	x	√
Sp1	x	x	√
W box	x	x	√
WUN	x	x	√

Supplemental Table 3. Analysis of promoter of CaTLP1 homologs. ABRE consensus sequence ACACGTGNC is shown for representative plant species.

Plant species	Motif
Glycine max	CACGT
Arabidopsis lyrata	ACGTG
Arabidopsis thaliana	ACGTG
Populus tricocarpa	ACGTG
Ricinus communis	CACGTC
Cicer arietinum (CaTLP1)	CCACGTC
Cicer arietinum (*CaTLP2*)	GCAACGTGTC
Sorghum bicolor	CACGT
Vitis vinifera	CACGT
Physcomitrella patens	CACGT

Supplemental Table 4. CaTLP1 interacting proteins identified from immunoprecipitated complexes.

Accession No.	Protein Name
gi|15218569	CK2; casein kinase I/ kinase [*Arabidopsis thaliana*]
gi|15224781	CKL5 (Casein Kinase I-like 5); casein kinase I/ kinase [*A. thaliana*]
gi|15236114	CK1 (casein kinase 1-like protein 1); casein kinase I/ kinase [*A. thaliana*]
gi|52077492	putative protein kinase ADK1 [*Oryza sativa* japonica] / predicted casein kinase I
gi|224116874	predicted protein [*Populus trichocarpa*]
gi|147859066	hypothetical protein [*Vitis vinifera*]
gi|168044758	predicted protein [*Physcomitrella patens* subsp. patens]
gi|223973105	unknown protein [*Zea mays*]
gi|224091248	predicted protein [*Populus trichocarpa*]
gi|225442217	predicted hypothetical protein [*Vitis vinifera*]

Supplemental Table 5. List of primers used in this study.

Primer Name	Primer Sequences (5′- 3′)
TubGSP1	ACCACCCCGCTTCGATTTACTACCTATT
TubGSP2	CACGGACTATGTTTTTCAATGGCAT
TubBaMF	CGCGGATCCATGCCATTGAAAAACATAG
TubSalR	ACGAGTCGACTCACAGGCTGGTTTAG
TUBBaM22F	CGCGGATCCGATGCCATTGAAAAACAT
TUBSaL22R	ACGCGTCGACTTCACAGGCTGGTTTA
CaTLP1GATEWAYCF	CACCATGCCATTGAAAAACATAGTCCGT
CaTLP1GATEWAYCR	CACAGGCTGGTTTAGTATCAAAGCTT
CaTLP1GATEWAYPF	CACCCTATGGTGTATATAAGTGG
CaTLP1GATEWAYPR	GTTTCCACACCTTACAAACTCC
AtTLP2RT-F	GAGAGAGGTTCAGGTTCGTTTTG
AtTLP2RT-R	CGCCGATCTCACCGTAGGT
CaTLP1RTF	GAGGAAGGAGGAAATGCTCCA
CaTLP1RTR	GTGAGGGAGAAAAAGGTTCGTC
RTPOLYUBIFP	TGCCGACTACAACATCCAGAAG
RTPOLYUBIRP	TGCATGCCTCCACGAAGAC
RTGROELFP	CGGTAAATCCGTCGCTCGTA
RTGROELRP	TCACCGTCGGGACTCTGAAG
M13FP	GTTTTCCCAGTCACGACGTTG
M13RP	TGAGCGGATAACAATTTCACACAG
M13F	CGCCAGGGTTTTCCCAGTCACGAC
M13R	AGCGGATAACAATTTCACACAGGA
GUS584F	AAGACTGTAACCACGCGTCTG
GUS626R	GTTCAACGCTGACATCACCA
RAB18-5L	TCGGTCGTTGTATTGTGCTTTTT
RAB18-7R	CCAGATGCTCATTACACACTCATG
RD20-REALF2	TTAGCTCCGGTCACCAGTCA
RD20-REALR2	CATGTATGGTTTTGGTAATGTTTCC
AtTLP2FP	ATGTCTTTGAAAAGCATCCTTCGTG
AtTLP2RP	TTACCCTTCACATGCCGGTTT
CaTDNALPEF	GGATAGGTCTGGTTTTCTGGG
CaTDNARPER	ATGATTGCCTTTGCATGACTC
CaTDNALPP1F	CCTTGTGATTGCGAATTTTTC
CaTDNARPP1P2R	AAGATCGGAAAGAGAAACGC
CaTDNALPP2F	ATATGCTTTCAAACCCCCAAC
EF-1α-qPCR-for	CTTGCTTTCACCCTTGGTGT
EF-1α-qPCR-rev	TCCCTCGAATCCAGAGATTG
CaCK1PENTRF	CACCATGGAACCTCGTGTTGGAAATAAG
CaCK1R	ATAATGGGTCCTCTCATCAGTCTCTAACTG
